# Magnetic and electrical properties of pseudowollastonite nanoceramic prepared by wet method

**DOI:** 10.1038/s41598-025-18717-0

**Published:** 2025-09-15

**Authors:** Z. A. Abd El-Shakour, Gehan T. El-Bassyouni, Esmat M. A. Hamzawy, Manal A. Mahdy, I. K. El Zawawi, H. H. A. Sherif, Gamal M. Turky

**Affiliations:** 1 Geological Sciences Department, National Research Centre, 33-El Buhouth St., Dokki, Giza, 12622 Egypt; 2 Refractories, Ceramics and Building Materials Department, National Research Centre, Dokki, Giza, 12622 Egypt; 3 Glass Research Department, National Research Centre, 33-El Buhouth St., Dokki, Giza, 12622 Egypt; 4 Solid State Physics Department, National Research Centre, 33-El Buhouth St., Dokki, Giza, 12622 Egypt; 5 Spectroscopy Department, National Research Centre, 33-El Buhouth St., Dokki, Giza, 12622 Egypt; 6 Microwave Physics and Dielectrics Department, National Research Centre, 33 El Bohouth St. (Former El Tahrir St.), Dokki, P.O. 12622, Giza, Egypt

**Keywords:** Pseudowollastonite nanoceramics, Structural properties, FTIR and RAMAN spectroscopy, Dielectric properties, Magnetic properties, Materials science, Physics

## Abstract

Pseudowollastonite with different CoO contents (0.0-9.5wt.%) was systematized by the wet precipitation method and sintered for 1 h at 1300 °C. XRD patterns demonstrated a nanoceramic composite structure with nanocrystallite sizes rising from 77.6 to 86.1 nm as CoO content increases from 0.0 to 9.5wt.% in composites. The addition of CoO causes RAMAN bands shift and intensity variations, indicating structural alterations. The magnetic moment values rise from 75.6 × 10^− 3^ to 0.481emu/g with increasing CoO content from 3.5 to 9.5wt.%. Hence the nanoceramics acquire a magnetic property from the CoO component. Electrode polarization dominates the spectra at lower frequencies. The dielectric spectra are conquered on the low frequency side by electrode polarization effects, although the investigated nanoceramics are insulators, and their resistivity rises with increasing CoO content. The higher frequency side is characterized by charge transport in a disordered matrix as the underlying physical mechanism. The novelty of this work lies on emerging pseudowollastonite phase via rising the sintering temperature to 1300 °C which opens applications for new industrial uses.

## Introduction

Silicates are a different type of bioceramic defined by a SiO_2_ network formation as well as network modifiers such as CaO, Na_2_O, P_2_O_5_, and/or MgO. Bridging oxygen connect adjacent silicate building units, which are referred to as Q_Si_^n^ (*n* = 0–4), with “n” denoting the number of these atoms in each unit^1^. However, the addition of network modifiers breaks the lattice by replacing bridging oxygen with non-bridging ones, dramatically affecting the characteristics of silicate bioceramics. Several articles have addressed the inclusion of dopants into silicate bioceramics. Furthermore, Co-doping reduces the grain size and increases the density of akermanite, resulting in higher microhardness and elastic modulus. Co-doping also slows the degradation rate while increasing apatite production^2^. Incorporating Co ions into borosilicate glasses, such akermanite [(Ca_2_Mg[Si_2_O_7_]), melilite mineral of the sorosilicate group], improves stability, angiogenesis, and bone formation without producing cytotoxicity^3^. The Co-dopants used in silicate bioceramics have specific properties. The advantage is that it promotes angiogenesis and has antimicrobial properties. Whereas it’s down sides are cytotoxic at large doses^4^.

According to Ali et al.^5^silica has a porous structure and cobalt (II) oxide (CoO) is an active material with several oxidation states. As a result, a composite made of cobalt oxide and silica would have interesting properties. It was found that silica is an excellent host material because it evenly distributes guest particles while impeding particle growth, which leads to the creation of highly dispersed nanocomposite materials. Extensive research has been conducted on cobalt silica composite materials for a variety of technological applications, including super capacitance^5^humidity, and gas sensing^7^and catalysis^6^.

As per Yu et al.^8^thermoelectric oxides exhibit greater interest in energy harvesting due to their superior high-temperature stability and general non-toxicity as compared to typical thermoelectric alloys. With its unusual misfit structure, which consists of an alternating sequence of conductive CoO_2_ layers and insulating rock salt layers along the c axis, calcium cobaltite (Ca_3_Co_4_O_9_) is a promising p-type oxide^9^. This unique structure leads to its intrinsically low heat conductivity. Yet, because of the layered crystal structure^10^ and the discrepancy between the thermal stability range of the Ca_3_Co_4_O_9_ phase and the eutectic point, the electrical properties of bulk Ca_3_Co_4_O_9_ materials are often constrained by inadequate densification.

Due to its ability to probe molecular fluctuations and charge transport in a broad frequency range, Broadband Dielectric Spectroscopy (BDS) is employed to study such electrical and dielectric features of the sample under investigations. It is used to probe charge transport, electrode polarization, and dynamic fluctuations in different contents of CoO in such rare mineral, pseudowollastonite. Further to show the effect of sintering temperature on the dielectric behavior. BDS measures the complex dielectric function, $\:{\epsilon\:}^{*}\left(\nu\:\right)=\:{\epsilon\:}^{{\prime\:}}\left(\nu\:\right)-i{\epsilon\:}^{"}\left(\nu\:\right)$, which is equivalent to the complex conductivity function, σ^∗^. This is expressed as: $\:{\epsilon\:}^{*}=\:\frac{{\sigma\:}^{*}}{i\omega\:{\epsilon\:}_{o}}$ and $\:{\sigma\:}^{*}=\:{\sigma\:}^{{\prime\:}}{\:+\:i\sigma\:}^{"}$, implying that $\:{\sigma\:}^{{\prime\:}}=\:{\epsilon\:}_{o}\omega\:{\epsilon\:}^{"}$ and $\:{\sigma\:}^{"}=\:{\epsilon\:}_{o}\omega\:{\epsilon\:}^{{\prime\:}}$ ($\:{\epsilon\:}_{o}$ being the vacuum permittivity and $\:\nu\:$ the frequency of the applied electric field).

This work aimed to study the impact of increasing CoO contents at Pseudowollastonite ceramic sintered at a high temperature of 1300 °C on the structure, surface morphology, FTIR spectra, and Raman spectroscopy, magnetic and electric properties of Pseudowollastonite with different CoO contents (0.0-9.5 wt%) for advanced magnetic and electric applications.

## Experimental

### Materials

The cobalt in pseudowollastonite was synthesized using a low-cost wet precipitation process. To obtain calcium nitrate Ca(NO_3_)_2_ solution, calcium carbonate [CaCO_3_, 99% El-Gomhorya Chemical Company, Cairo Egypt] was dissolved in a sufficient volume of concentrated nitric acid. Silica gel [Dioxosilane, Fluka, Germany] was used as a precursor for SiO_2_. During the preparation procedure, varying volumes of cobaltous acetate [Co(CH_3_CO_2_)_2_·4 H_2_O, BDH Limited Poole, England] were used to provide CoO_2_. In the current study, there are four weight% concentrations. The amounts produced were 0.5, 3.5, 6.5, and 9.5 g of cobalt oxide in 100% Pseudowollastonite slurry. To ensure thorough mixing and homogeneity, the gel was aged and continually magnetically stirred. The gel was dehydrated at 100 °C for 24 h, and then the fine particles was dried and made into discs with a 20 KN uniaxial pressure. The precursor was sintered at 1300 °C for an hour, ground into powder with a ball mill, and sifted through a 63 μm standard sieve. A series of cobalt-rich calcium silicate samples were analyzed using XRD, FTIR, RAMAN, SEM, and VSM.

Table 1 displays the sample composition, the code for the composition, and the quantity of cobalt oxide added.

**Table 1:** Sample compositions and symbolization.

Sample code	Composition in wt%		Additive over 100% composition in oxides in g
	CaO	SiO_2_	CoO
PC0.0	48.28	51.72	–
PC0.5	31.01	48.53	0.5
PC3.5	31.01	48.53	3.5
PC6.5	31.01	48.53	6.5
PC9.5	31.01	48.53	9.5

This preparation procedure is identical to that utilized in the previous work^11^but there is a fundamental change reflected by raising the annealing temperature to 1300 °C in the work under study.

### Characterization

X-ray diffraction examination was performed on powder samples using an Empyrean diffractometer system (XRD, model Bruker D8, Germany) with Cu-K_α_ radiation at λ = 0.15418 nm to analyze their crystalline phases. The 2θ range was assessed by XRD between 15 and 80°, to evaluate the produced samples’ purity, phase development, and size of crystalline particles^12^.

The materials’ microcrystalline structure and surface morphology were determined using a field emission scanning electron microscope (FE-SEM, model FEG Quanta 250 FEI, Netherlands) operating at 15 kV. To improve scanning images, a layer of gold was added to the sample surfaces prior to SEM examinations. FTIR spectroscopy was utilized to investigate structural and chemical features.

FTIR spectroscopy was utilized to investigate structural and chemical features. At room temperature, FTIR analysis was carried out with a Type A FT/IR- 6100 spectrometer (Jasco, USA) that had a spectra resolution of 4 cm^− 1^ and a vibrational wavenumber range of 400–1600 cm^− 1^.

An i-Raman Plus 532 S portable laser Raman spectrometer equipped with a BAC151C Raman microscope inspection system (B & W TEK, USA) with 20-100X lenses was used to assess the materials’ Raman shift^13^.

The electrical and dielectric properties of current samples are performed via a powerful broadband dielectric spectrometer, BDS, utilizing a high resolution Alpha-analyzer with an active sample head–Novocontrol, GmbH concept 40, Germany. All measurements were investigated on a broad range of frequency ranging from 0.1 Hz to 20 MHz) and at ambient temperature. The disc sample was sandwiched between two gold-coated brass electrodes with a diameter of 10 mm in parallel plate geometry. WINDETA software handled the control and data collecting activities^14–16^. The magnetic properties of the sample under investigation were checked by using vibrating sample magnetometer (VSM-7410, Lakeshore, USA).

## Result and discussion

### Structural analysis

**Fig. 1:**
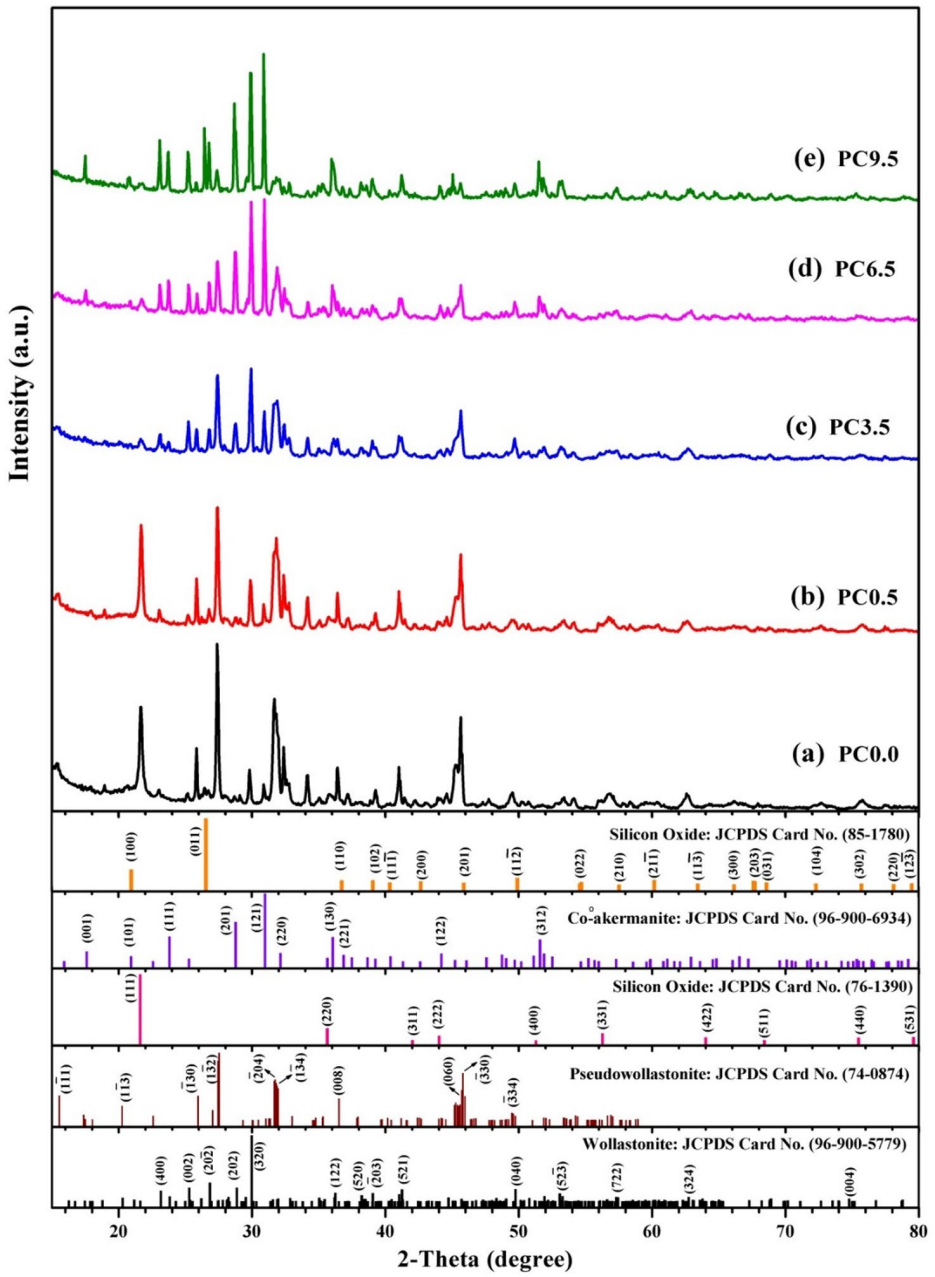
X-ray diffraction patterns of composites sintered at 1300 °C (**a**) PC0.0, (**b**) PC0.5, (**c**) PC3.5, (**d**) PC6.5, and (**e**) PC9.5.

The internal structure of the pure and cobalt oxide doped samples that were sintered at 1300 °C for one hour is inspected by X-ray diffraction (XRD) patterns, as illustrated in Fig. 1. The XRD pattern in Fig. 1a for pure powder (WC0.0) reveals that there are three structural phases. The main phase of these three crystalline phases is a triclinic phase of Pseudowollastonite that is well-matched with the international diffraction card (Ca_3_(Si_3_O_9_, ICDD # 96- 74-0874). The other two phases are the monoclinic and cubic phases of wollastonite and silicon oxide, which are consistent with the international diffraction cards (CaSiO_3_, ICDD # 96-900-5779) and (SiO_2_, ICDD # 96-76-1390) respectively, as shown in Fig. 1. The highest diffraction peaks of the identified three phases of triclinic, monoclinic, and cubic are positioned at 2θ = 27.53°, 29.97°, and 21.6°, respectively. In the same way, the WC0.5 sample is crystallized in the same three crystalline phases: triclinic, monoclinic, and cubic phases, in addition to the tetragonal phase of Co-åkermanite structure, as shown in Fig. 1b. The tetragonal phase of Co-åkermanite structure is well corresponded to the international diffraction card no. (Ca_2_CoSi_2_O_7_, ICDD # 96-900-6934), with the main diffraction peak found at 2θ = 30.97°.

Also, the internal structure of samples with cobalt oxide concentrations of 3.5 wt% and 6.5 wt% (PC3.5 and PC6.5) is identified by three phases of triclinic, monoclinic, and tetragonal for pseudowollastonite, wollastonite, and Co-åkermanite structures, respectively (Fig. 1c, d). However, the sample with the highest cobalt oxide concentration of 9.5 wt% (PC9.5) is identified by the same phases of triclinic, monoclinic, and tetragonal, besides the appearance of the hexagonal phase of the SiO_2_ phase, as clarified in Fig. 1e. The Co-åkermanite phase structure increases with increasing cobalt oxide addition in the sample, and it becomes the main phase in samples PC6.5 and PC9.5 of the highest CoO concentrations (Fig. 1d, e).

From the current structure discussion, it could be observed that the sintering temperature and cobalt oxide concentration are considered the important factors for changing the internal structures of the prepared nanoceramic composites. The calcination at 1300 °C in this work has mainly changed the internal composite phases compared to that heat treated at 1000 and 1200 °C that were previously declared in the previous work^11^. The change of the phase from wollastonite to pseudowollastonite via increasing the sintering temperature to 1300 °C for the same sintering time led to a change in nanoceramic properties and applied applications.

The crystallite size (C_S_) of the pure and doped composite is inspected from the highest intensive peak of each XRD pattern according to the following equation^17^:

1 $${{\text{C}}_{\text{S}}}={\text{k}}\lambda /\beta {\text{cos}}(\theta )$$

C_S_ denotes the crystallite size, β symbolizes the peak broadening at half maximum, θ is the diffraction angle, and k is the shape factor. The calculated crystallite size (C_S_) for the powder samples calcined at 1300 °C are 77.6, 71.8, 84.2, 86.1, and 86.1 nm. It was noted that the CS generally increased with increasing the concentration of cobalt oxide up to PC6.5 and then remain constant at PC9.5. This stability could occur because the Co-åkermanite phase has already achieved its maximum thermodynamic stability and growth rate at 6.5 wt%, and the additional CoO in PC9.5 primarily contributes to the formation of minor phases (e.g., hexagonal SiO_2_) rather than further enlarging Co-åkermanite crystallites^18^.

### Surface morphology

**Fig. 2:**
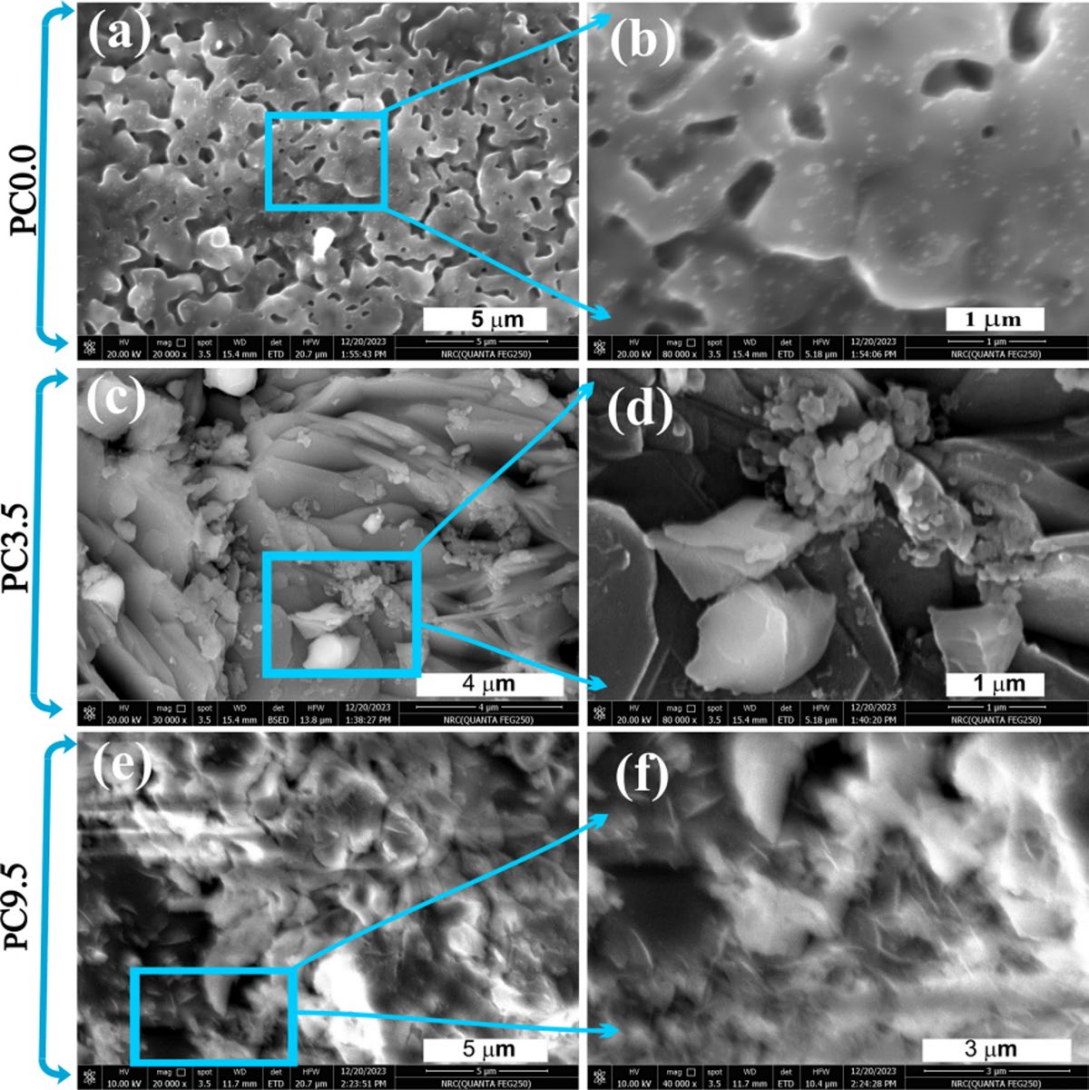
FE-SEM micrographs of (**a**,**b**) PC0.0, (**c**,**d**) PC3.5, and (**e**,**f**) PC9.5 samples sintered at 1300 °C.

The surface morphology of the samples has been identified by the field emission scanning electron microscope (FE-SEM), as seen in Fig. 2. As all samples under investigation have been heat-treated to a high temperature of 1300 °C, it has caused the internal nanoparticles to accumulate into big grains, as seen in Fig. 2. For the un-doped sample (PC0.0) in Fig. 2a, b, the grains are connected from one or two sides, forming a net in which hardly any nanoparticles are seen. In Fig. 2b, some nanoparticles are observed on the surfaces of grains. The nanoparticles observed on the surfaces of grains (Fig. 2b) are those nanoparticles which could not be included in the accumulated grains and still outside on their surfaces.

At Fig. 2c, d for sample (PC3.5) with CoO content, most of the nanoparticles are accumulated in elongated grains to about 3 μm length, and still some nanoparticles are observed at Fig. 2d. At Fig. 2e,f for the highly doped sample of CoO (PC9.5), the FE-SEM micrographs are unclear but declare that the grains are mostly connected, but some nano-pins are seen, probably due to the magnetic phase of Co-åkermanite (Ca_2_CoSi_2_O_7_).

### FTIR spectroscopy

**Fig. 3:**
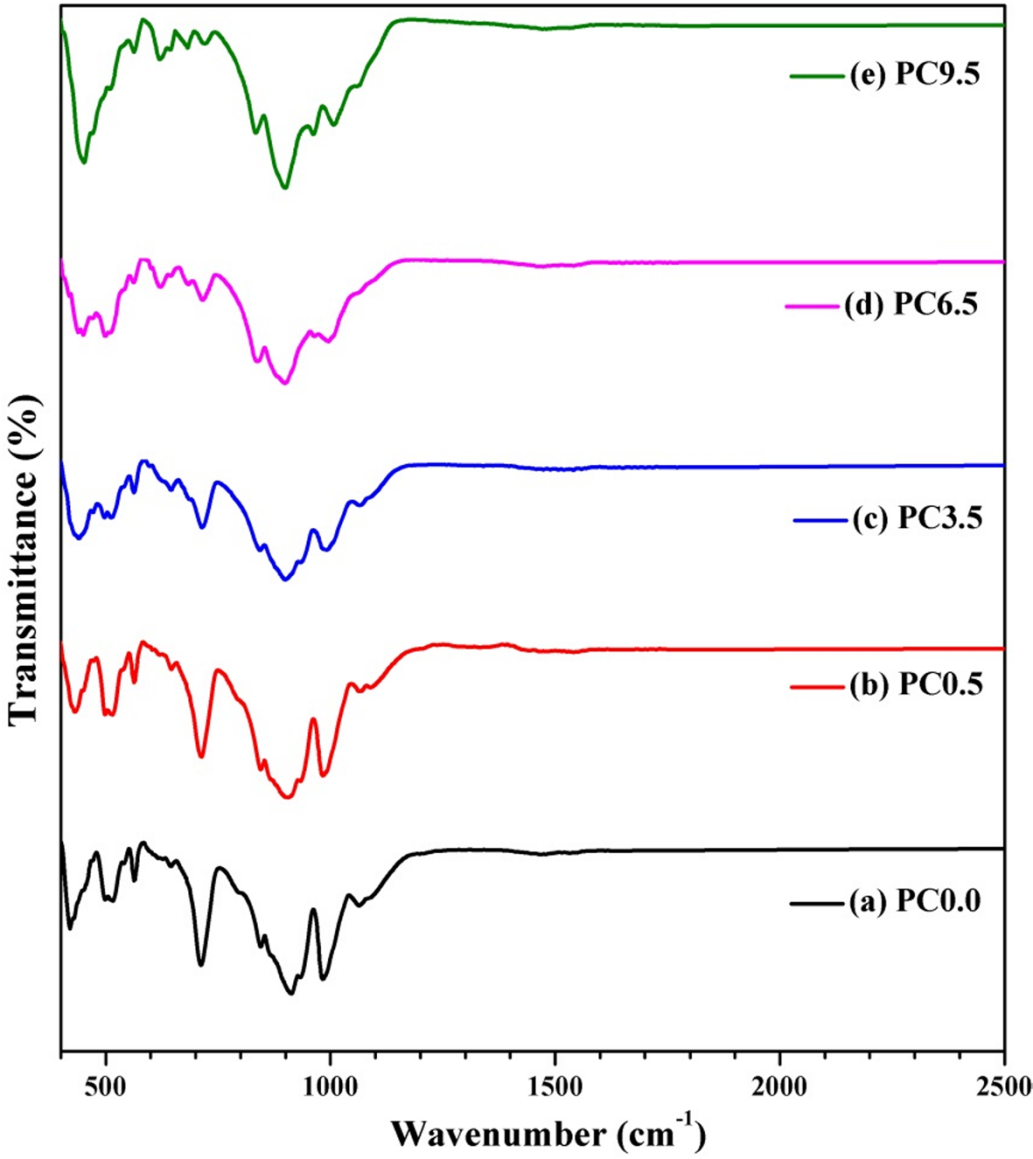
The FTIR spectra of the nanoceramics with indicated CoO content sintered at 1300 °C.

Figure 3 depicts the FTIR spectra of Ca_3_(Si_3_O_9_)/CoO samples sintered at 1300 °C, showcasing variable concentrations of CoO. Table 2 provides the FTIR assignments for Ca_3_(Si_3_O_9_)/CoO powder heat-treated at 1300 °C. As the CoO content increases, noticeable changes are observed in the samples. Si-O stretching vibrations are perceived at 1089, 1063, 1007, and 931 cm^− 1^, as reported in previous studies^19–21^with distinct variations attributed to the presence of CoO.

The peak corresponding to Si-O stretching vibrations at 1089 cm^− 1^ disappears at CoO contents higher than 0.5%. The medium peak at 1063 cm^− 1^ disappears at higher CoO concentration (6.5% and 9.5%). Likewise, the peak at 1008 cm^− 1^ disappears, being substituted by two peaks at 994 cm^− 1^ and 964 cm^− 1^ at 6.5% CoO content. These peaks then shift to 1007 cm^− 1^ and 962 cm^− 1^ at 9.5% CoO content. Additionally, the peak at 931 cm^− 1^ disappears with the addition of CoO.

The Si-O-Ca stretching vibration is seen at 913 cm^− 1^ and 845 cm^− 1^^22^. The peak at 913 cm^− 1^ undergoes a red shift to 898 cm^− 1^, while the peak at 845 cm^− 1^ experiences a blue shift to 834 cm^− 1^. Additionally, the Si-O vibration, represented by a peak at 712 cm^− 1^, shifts to a higher wavenumber, reaching 721 cm^− 1^, and its intensity decreases with increasing CoO content. In samples with a higher CoO content of PC6.5% and PC9.5%, the bending vibration of Si-O-Si bonds is detected at 683 cm^− 1^. While O-Si-O bending vibration is seen at 645 cm^− 1^^21^, another bond emerges at 620 cm^− 1^ at higher CoO concentrations. The peak at 565 cm^− 1^ faded from a determinedly sharp peak to a weak shoulder, while at 512 cm^− 1^ the peak disappeared. The peaks corresponding to the Si-O-Si bending vibrations are observed at 499 cm^− 1^^20,23^. Meanwhile, the peak representing the O-Si-O bending vibrations at 420 cm^− 1^ undergoes a blue shift to a higher wavenumber, eventually reaching 452 cm^− 1^.

**Table 2:** FTIR assignment of Ca_3_(Si_3_O_9_)/CoO powder heat treated at 1300 °C.

Assignment	References	Base 1300	0.5%	3.5%	6.5%	9.5%
Si–O stretching vibration	^19–21^	1089	1089	–	–	–
		1063	1063	1064	–	–
		983	983	992	994	1007
		–	–	–	964	962
		933	–	–	–	–
Si-O-Ca stretching vib.	^22]]^	913	905	900	898	898
		845	845	843	838	834
Si-O- vib.	^22]]^	712	713	714	715	721
Si-O-Si bending vib.		–	–	–	683	681
O-Si-O bending vib.	^21]]^	645	645	645	644	644
		–	–	–	621	620
		563	563	562	562	562
Si-O- Si bending vib.	^20,23^	499	499	497	498	
δ3(O − Si − O)	^20,23^	420	431	440	450 439	452

### Raman spectroscopy

**Fig. 4:**
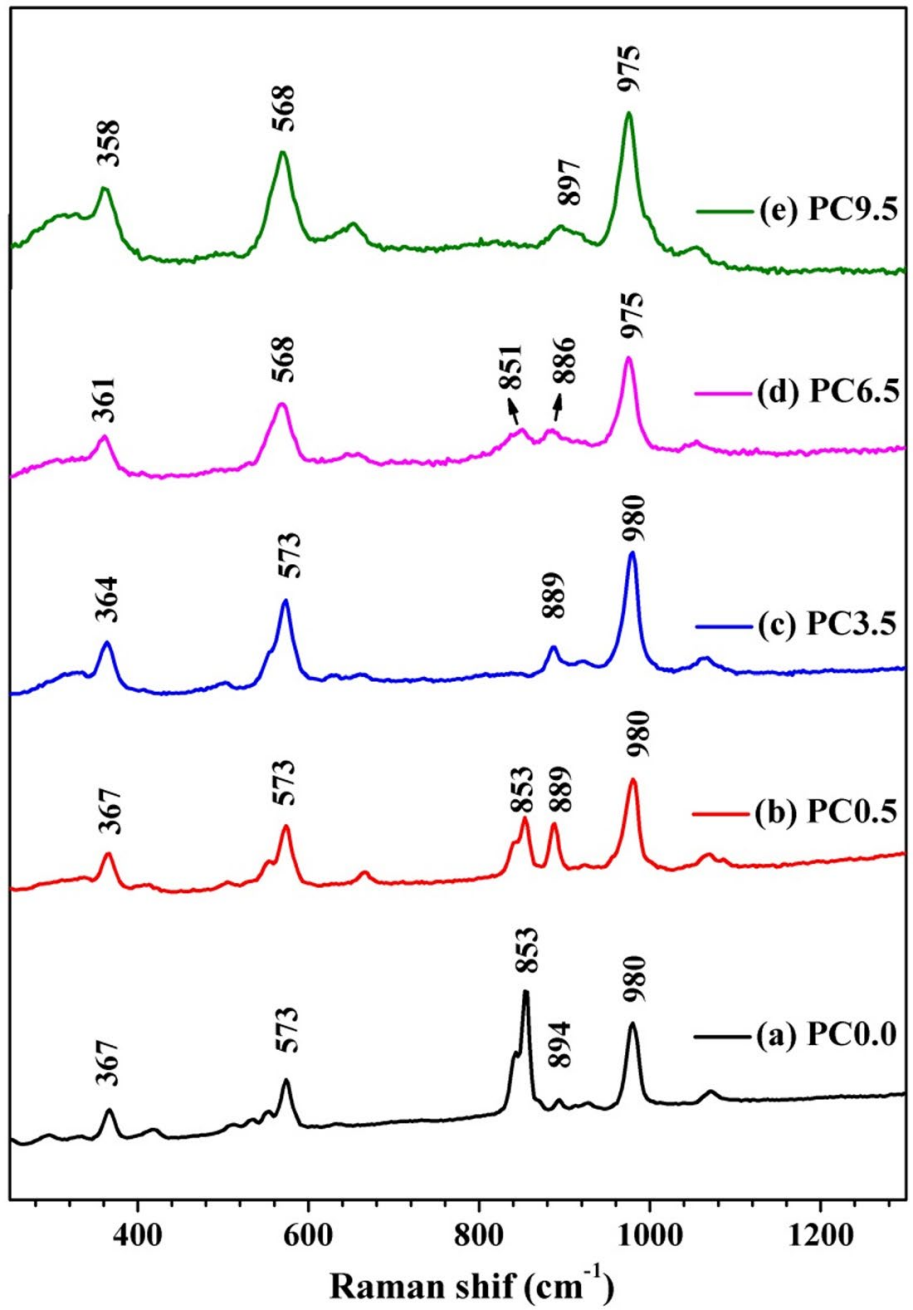
RAMAN spectra of the prepared nanoceramics of indicated CoO content heat treated at 1300 °C.

Raman spectroscopy was utilized to investigate the influence of CoO addition on the prepared Ca_3_(Si_3_O_9_). The Raman spectra of Ca_3_(Si_3_O_9_)/CoO, depicted in Fig. 4, reveal significant Raman shift bands at 980, 894, 853, 633, 573, as well as 367 cm^− 1^. The 980 cm^− 1^ band indicates the asymmetric stretching of Si-O^24^, while the 894 cm^− 1^ and 853 cm^− 1^ bands represent the symmetric stretching vibration of Si-O^24–26^. The bands at 633 cm^− 1^ and 573 cm^− 1^ are apportioned to Si-O-Si symmetric bending vibration^24–27^ and the lattice vibrations of Ca-O are evident at 367 cm^− 1^^24,26^.

The bands at 980, 573, and 367 cm^− 1^ change to shorter wavelengths with the addition of CoO. The bands at 894 cm^− 1^ and 633 cm^− 1^ also showed a substantial shift towards lower wavelengths upon the addition of CoO, followed by a smaller shift towards higher wavelengths in the highest concentration of CoO. Additionally, the intensity of the symmetric stretching of the Si-O band at 853 cm^− 1^ decreases until it disappears at the highest concentration of CoO (9.5%).

In conclusion, the Raman spectroscopic analysis of Ca_3_(Si_3_O_9_)/CoO samples revealed significant changes in the Raman shift bands upon the addition of CoO. The observed shifts and intensity changes in the Raman bands, such as the asymmetric and symmetric stretching of Si-O, Si-O-Si symmetric bending vibration, and Ca-O lattice vibrations, suggest structural modifications induced by the addition of CoO and its varying concentrations.

### Electrical investigations

**Fig. 5:**
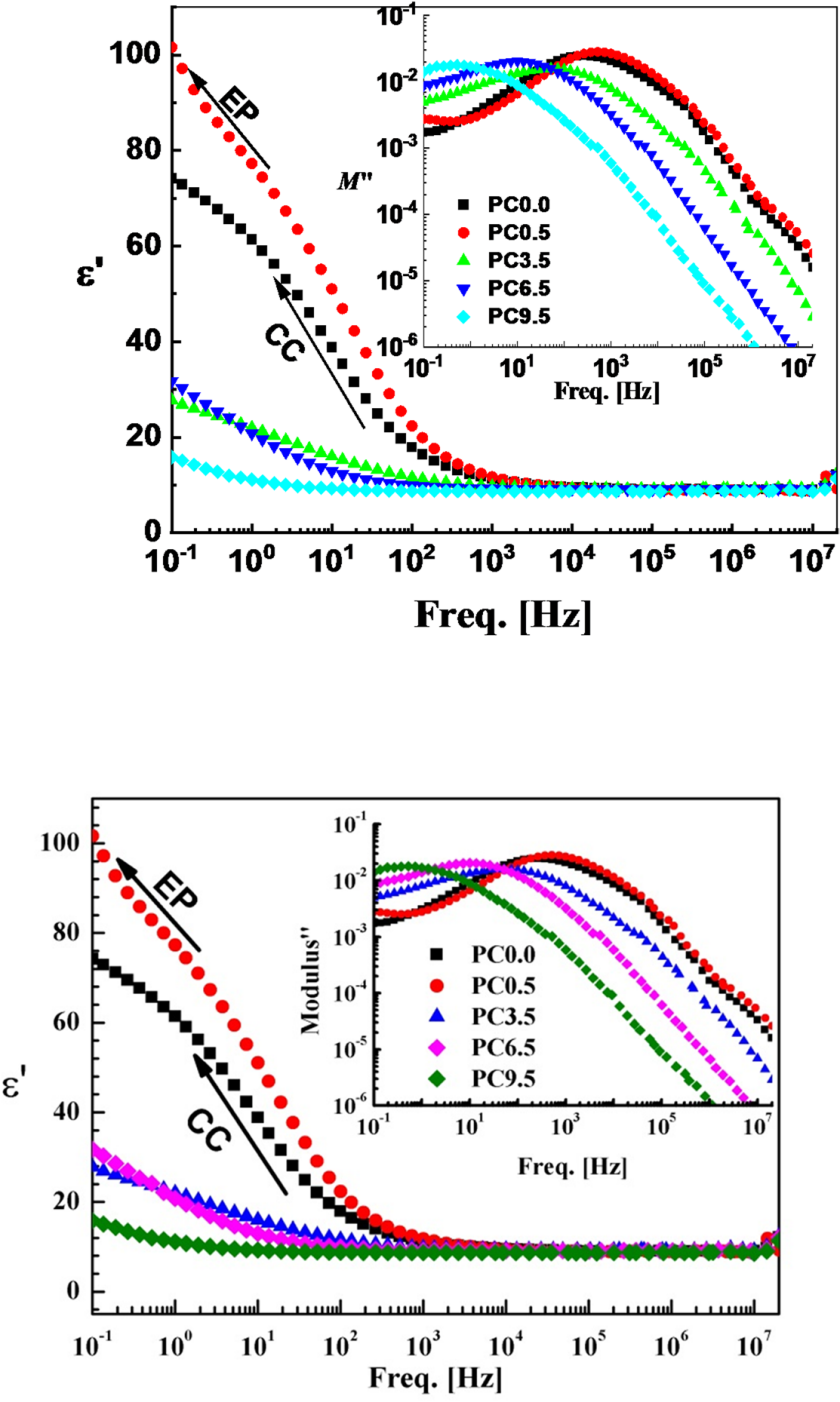
Permittivity as determined at ambient temperature versus frequency of heat treated at 1300 °C Ca_3_(Si_3_O_9_)/CoO nanoceramics of different CoO content as indicated. It is reflecting clearly the effect of both conductivity contribution, CC, and electrode polarization, EP. Inset shows similar presentation of the electric loss modulus.

The dielectric and electrical properties of the investigated samples were achieved using a broadband dielectric spectroscopy in broad frequency range (0.1 Hz – 20 MHz) and at room temperature. The permittivity, ε′, is presented against frequency in Fig. 5 for the prepared Ca_3_(Si_3_O_9_)/CoO of different CoO concentrations that heat treated at 1300 °C. At higher frequencies starting from 1 kHz, there is no remarkable, effect neither of frequency nor of the composition, on the permittivity of the composite can be noticed. This behavior is now considered as a common feature in many dielectric materials^28–32^. At relatively higher frequencies, the long-range hopping mechanism of the free charge carriers and/or the interfacial polarization oscillation lag behind the applied electric field alteration. This is why no effect of composition as well as the frequency of the external field on permittivity could be seen at such higher frequencies.

Further decrease of frequency from 1 kHz down to 0.1 Hz shows increasing and splitting up of the permittivity, ε′, according to frequency and composition. One has to notice that the permittivity generally and remarkably reduced as the CoO concentration increases. This can be explained taking into consideration the fact that, accumulation of the nanoparticles into big grains with increasing CoO content under the effect of heat treatment that detected previously by the field emission scanning electron microscope (FE-SEM) attenuated remarkably the dynamics of both interfacial polarization and charge carriers’ transport as well and hence reduced the permittivity.

The electric loss modulus (M″) as a function of frequency is displayed in the inset of Fig. 5. Since it presents the dynamics under consideration as peaks and shields them from common problems like electrode nature, surface-contact and space-charge injection phenomena. Taking in to consideration also the conduction contribution (CC) usually masked up the relaxation processes in the dielectric spectra. The representation M″(ν) has made it easier to understand the dynamic relaxation of various processes. Since it offers a clear picture of DC-conduction and dipole relaxation, the dielectric loss modulus representation is employed in dielectric characterization^33–35^. There is a relationship between the dielectric loss ε″ and the electric loss modulus, or the imaginary component of the complex modulus, M” as follows:

2 $$M^{\prime\prime}=\frac{{\varepsilon ^{\prime\prime}}}{{{\varepsilon ^{\prime 2}}+{\varepsilon ^{\prime \prime 2}}}}$$

The main peak shown in the electric loss modulus as presented against frequency in the figure’s inset, originated from the dc-conductivity contribution. It shifts remarkably towards lower frequencies as the CoO concentration increases. This reflects the elongation of the hopping time of the free charge carriers that will cause the reduction of dc-conductivity. It is very interesting to show a small peak at the lowest frequencies, especially for both PC0.0 and PC0.5 samples accompanied by a clear change in the trend of permittivity. The question raised here if there is electrode polarization (EP) effect in such electrical insulator compositions (that originated from accumulation of free charge carriers at the metal electrodes).

**Fig. 6:**
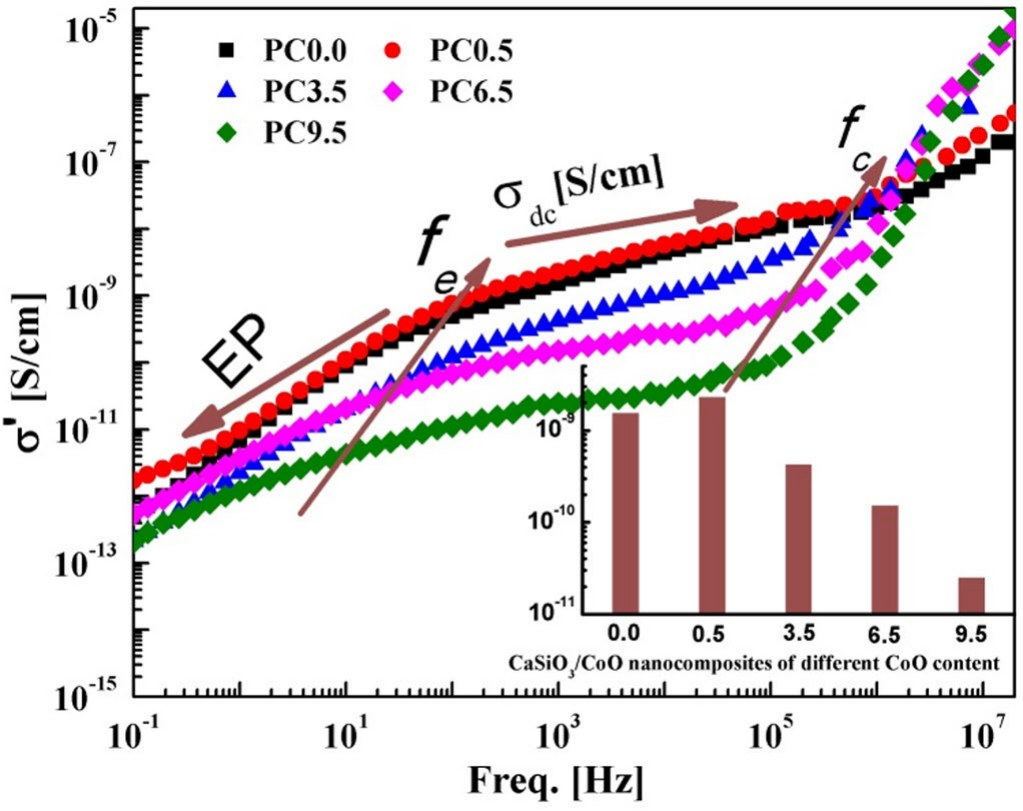
The ac-conductivity as determined at ambient temperature versus frequency of heat treated at 1300 °C Ca_3_(Si_3_O_9_)/CoO nanoceramics of different CoO content as indicated. The inset shows the dc-conductivity of investigated samples deduced from the figure.

In order to study the effect of adding CoO on the electrical properties of the Ca_3_(Si_3_O_9_), the ac-conductivity,σ′, determined for the prepared samples and illustrated graphically as a function of frequency as signified in Fig. 6.

The figure illustrates how ac-conductivity, or the real part of the complex conductivity σ′, is characterized by a plateau-like behavior over the intermediate frequency range. The dc-conductivity, σ_dc_, is directly considered by the value of ac-conductivity of the plateau. A characteristic frequency, *f*_c_, at which dispersion begins and takes the form of a power law, is displayed in the higher frequency window. Interfacial effects, which result from the impediment of charge carriers by impenetrable walls at the external electrodes touching the sample, become predominant in the conductivity/dielectric spectra at lower frequencies (1 kHz down to 0.1 Hz). This is why the conductivity decreases graduallystarting from some definite frequency, *f*_*e*_, as the frequency decreases, and this phenomenon became a main feature in many highly conducive materials, e.g., polymers, nanocomposites, ionic liquids, and conductive hydrogel^36–41^ reflecting the effect of electrode polarization, which has led to a tremendous increase in both parts (real and imaginary) of complex permittivity at lower frequencies.

Electrode polarization, EP, is associated with a change in the well-known Electrical Double Laye charge density at the electrode- electrolyte (or ion conductor) interface. Charge transport and electrode polarization in conductive systems are highly technologically important subjects in contemporary research.

The inset of Fig. 6 illustrates the determined dc-conductivity of the pure and cobalt oxide doped samples that were sintered at 1300 °C for one hour. The conductivity determined at 1 kHz is considered to be the dc-conductivity and for all samples is relatively low. It is for the pure nanoceramic (PC0.0) 1.5 × 10^− 9^ S/cm and for the sample PC0.5 tends to be 2.29 × 10^− 9^ and then reduced gradually as the CoO concentration increases (3.5–9.5 wt%) to be 4.2 × 10^− 10^, 1.5 × 10^− 10^, and 2.47 × 10^− 11^ for the nanoceramics PC3.5, PC6.5, and PC9.5, respectively. This means that we have here a very strange behavior, how such phenomenon that characterizes the conductive polymeric systems and ionic liquids raised here in such insulator nanoceramics. The reduction of conductivity with increasing Co is due to the accumulation of charge carriers at the metal electrode originating the so-called electrode polarization, even its conductivity is remarkably higher that of the matrix.

### Magnetic analysis

**Fig. 7:**
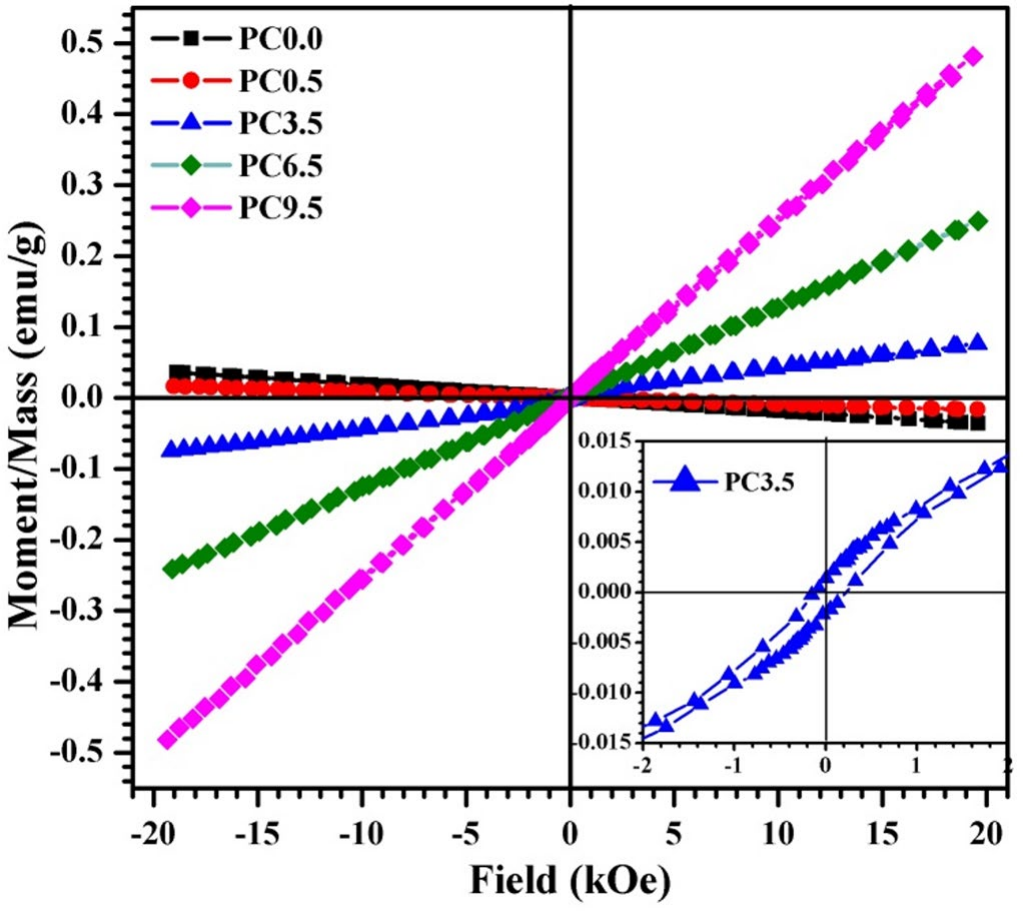
M-H curves of the prepared nanoceramics of various CoO content heat-treated at 1300 °C.

The room temperature magnetic characteristics of the produced composites with varying cobalt oxide concentrations that were sintered at 1300 °C for one hour are shown in Fig. 7, and a difference in magnetic behavior for each sample with respect to others was observed. Both the pure composite (PC0.0) and that with the lower concentration of cobalt oxide (PC0.5) do not have magnetic properties since they exhibit the same diamagnetic behavior. Worthily, it is clear that their values ​​are close to each other, and this means that the small percentage of cobalt oxide did not provide a magnetic property to the sample. However, other concentrations of cobalt oxide (3.5–9.5%) provide a magnetic behavior to the prepared composites, as shown in Fig. 7. This magnetic behavior represents a paramagnetic that clearly appears for nanoceramics PC3.5, PC6.5, and PC9.5. Upon closer examination of the magnetic behavior, it was found that both PC6.5 and PC9.5 nanoceramics have a linear curve that verified a paramagnetic behavior. While, PC3.5 nanoceramic has two magnetic contributions: paramagnetic contribution that is shown in the high applied field (> 2 kOe) and the weak ferromagnetic contribution, which is seen in the lower field (< 2 kOe) as displayed in the inset figure (Fig. 7) since the curve linearity is not verified. The weak ferromagnetic contribution has a low coercive field of 169.6 Oe and remanence of 1.66 × 10^− 3^ emu/g. It is also worth mentioning that as the concentration of cobalt oxide in the examined nanoceramics increases, so do the magnetic moments. They are 75.6 × 10^− 3^, 0.245, and 0.481 emu/g for nanoceramics PC3.5, PC6.5, and PC9.5, respectively; the higher value of magnetic moment is shown at 9.5 wt%.

According to Moriya^42^ and Barbar et al.^43^the Dzyaloshinskii-Moriya interaction caused the finding of weak ferromagnetic behavior in antiferromagnetic states. Also, Kothari et al.^44^and Park et al.^45^stated that the weak ferromagnetic behavior affected by the size effect that originated by spin canting. The calcination temperatures of 1000 and 1200 °C in the previous work^11^ had an impact on the magnetic data; this could be because they revealed a change in the internal crystalline structure. Ironically, when compared to earlier work^11^we discover that the paramagnetic behavior is prominent in both the current study after sintering at 1300 °C and the previous one sintered at 1000 °C. But, we see a significant difference in magnetization values between the two works. Magnetization values increased up to 0.195 emu/g during sintering at 1000 °C, while in the current study, they increased to 0.481 emu/g at the maximum CoO concentration. This is certainly attributed to the change in the internal structure, since at 1000 °C, the material was wollastonite (CaSiO_3_), but at 1300 °C, it changed to pseudowollastonite Ca_3_(Si_3_O_9_). The produced nanoceramics comprise a Co-åkermanite phase (Ca_2_CoSi_2_O_7_) which characterized by a reversible linear M-H curve as previously reported^43^. As a result, it is regarded as a source that gives magnetic characteristics to the resulting nanoceramics, even at the expense of other constituent phases.

We conclude from the previous discussion that the non-magnetic pseudowollastonite-based nanoceramic can acquire magnetic characteristics by including cobalt oxide. Because of the Co-åkermanite structure, adding CoO to the pseudowollastonite-based composite resulted in the creation of a novel “magnetic property” in nanoceramic materials. Adding cobalt oxide up to 9.5 weight% improves the magnetic moment of this magnetic characteristic.

## Conclusion

By wet precipitation route, Co-containing (0.0–9.5 wt%) pseudowollastonite compositions were prepared. Through a high sintering temperature process at 1300 °C, novel nanoceramics were obtained. The structural analysis proves the nanocomposite of crystallite nanoparticle ≤ 86.1 nm, which has three structural phases: the triclinic phase of pseudowollastonite (Ca_3_(Si_3_O_9_)); the other two phases are the monoclinic and cubic phases of wollastonite (CaSiO_3_) and silicon oxide (SiO_2_) respectively, in addition to the tetragonal phase of Co-åkermanite (Ca_2_CoSi_2_O_7_) structure for samples doped with CoO ≥ 0.5 wt%. The surface morphology showed that nanoparticles accumulated due to high sintering temperatures, shaping ellipsoidal grains seen by FE-SEM micrographs. FTIR spectroscopy showed some changes at higher CoO content (6.5% and 9.5%); the bending vibration of Si-O-Si bonds is observed at 683 cm^− 1^, and another bond emerges at 620 cm^− 1^, while the peak at 512 cm^− 1^ disappears. The increase of crystallite size (Cs) with the cobalt oxide quantity results in significant variations to the Raman shift bands. Also, the shifts and intensity variations seen in the Raman bands indicate structural changes caused by the addition of CoO at different concentrations. The Raman spectroscopic analysis showed changes in the Raman shift bands for the asymmetric and symmetric stretching of Si-O, Si-O-Si symmetric bending vibration, and Ca-O lattice vibrations after increasing the CoO content. Adding cobalt oxide to non-magnetic pseudowollastonite nanocomposite improves the magnetic moment and magnetic characteristics gradually, especially at 9.5 wt% of CoO content. On the other hand, the increase of the CoO content remarkably reduced the electric conductivity of the investigated composites. It is reduced from 1.5 × 10^− 9^ S/cm for the CoO-free sample to be 2.47 × 10^− 11^ S/cm for the nanoceramics PC 9.5 (i.e. at 9.5 wt% of CoO content). Even though the investigated samples show the electrode polarization effect.

That ubiquitous phenomenon takes place at the interface between a metallic electrode and electrolyte and results in an increase by many orders of magnitude in the net dielectric response of the sample. This is attributed to the hindering effect of charge transport due to the presence of the electrodes used. Charge transport and electrode polarization in disordered systems are topics of special technological importance in contemporary science. The novelty of this work lies in the emerging of the pseudowollastonite phase as the sintering temperature rises up to 1300 °C for the first time, which will change the nanoceramic properties and open new opportunities for novel applied applications as antimicrobial and numerous technological industrial uses for its specific electric and magnetic properties.

## Data Availability

All data generated or analyzed during this study are included in this manuscript.
